# Retrospective IP Address Geolocation for Geography-Aware Internet Services

**DOI:** 10.3390/s21154975

**Published:** 2021-07-22

**Authors:** Dan Komosny

**Affiliations:** Faculty of Electrical Engineering and Communication, Department of Telecommunications, Brno University of Technology, 61600 Brno, Czech Republic; komosny@vut.cz; Tel.: +420-54114-6973

**Keywords:** database, location, geotargeted content, cybercrime, RIPE Atlas, MaxMind

## Abstract

The paper deals with the locations of IP addresses that were used in the past. This retrospective geolocation suffers from continuous changes in the Internet space and a limited availability of past IP location databases. I analyse the retrospective geolocation of IPv4 and IPv6 addresses over five years. An approach is also introduced to handle missing past IP geolocation databases. The results show that it is safe to retrospectively locate IP addresses by a couple of years, but there are differences between IPv4 and IPv6. The described parametric model of location lifetime allows us to estimate the time when the address location changed in the past. The retrospective geolocation of IP addresses has a broad range of applications, including social studies, system analyses, and security investigations. Two longitudinal use cases with the applied results are discussed. The first deals with geotargeted online content. The second deals with identity theft prevention in e-commerce.

## 1. Introduction

IP geolocation is a fundamental part of many Internet services and applications. It delivers the geographical location of any Internet device, independent of its use, installation, software, and hardware. Any of these locations may be needed retrospectively when the reason to locate the device was not known before, or when the locations were obtained but not archived. These usages include evaluation of longitudinal studies, observation of long-term location patterns, replication of past system states, study of long-term evolution of the global Internet, and investigation of crime incidents. In theory, there is an unlimited history of all IP address locations available. However, this is in reality not true as only pieces of historical data are available, which makes the retrospective location a challenge.

This work presents results of retrospective location. It also introduces an approach to handle missing historical data. The usage of the results is demonstrated by two longitudinal use cases. The first deals with the geotargeted online content in which some pages with dynamically generated content based on the viewers’ locations do not work for unknown reasons. The past viewers’ locations are used to investigate the reasons for the page loading errors. The second use case discusses the application in identity theft prevention. The history of address locations is used to estimate the confidence of the user travel between places of subsequent logins. In a secured system, such as an e-shop with stored credit card details for one-click payments, a confident suspicion of ID theft can prevent the payment to minimize fraud losses and chargebacks.

Historical IP geolocation databases are used to obtain past locations. Such databases are populated by various techniques, which include location self-reporting [1,2], network measurements [3,4], mining web content [5], host and domain-name analyses [6,7], and custom submissions [8]. The stored locations in a database are shared by a range of addresses to maximize the Internet space coverage.

I work with the historical ground truth that includes past IPv4 and IPv6 addresses and their locations over five years. There are approx. 51 k IPv4 and 17 k IPv6 addresses in the ground truth. This ground truth was linked to historical geolocation databases to observe the past address locations. The NA (not available; not returned) locations are also considered in the analysis as their number significantly affects the accuracy, especially for IPv6 addresses. The results show how far into the past the system may locate an address without a noticeable accuracy loss. I also work with the location lifetime in a database. For this purpose, I evaluate the database-stored location history of one year of about 421 k IPv4 and 47 k IPv6 addresses. Due to missing data, survival analysis is applied to process the data. Specifically, interval censoring is used to cover the past dates where the historical geolocation databases are missing. The fitted lifetime model allows for a given address to estimate the time when its location was changed in the past.

The presented results have a defined scope. The results dealing with retrospective IP address locations (Section 5) are limited to fixed devices. In addition, the historical ground truth is not evenly distributed across the world. Therefore, these results are mostly descriptive of Europe and North America. The results dealing with IP address location lifetime (Section 6) are also valid for mobile devices.

The analyzed locations were observed via the past MaxMind GeoLite2 City databases. This database is commonly shipped with various software and operating systems. This makes their past versions available in data archives such as OS repositories and software development snapshots on GitHub and GitLab. To the best of my knowledge, it is the only universal solution for retrospective location.

The historical data used in this work are made available as a single collection for result replication and further work in [9], with the list of files and their format described in Appendix A. The processed data are also published, including the observed past locations and lifetime durations. The particular sources used for obtaining the past databases are described.

The work is structured as follows: The general problem of missing past IP geolocation databases is discussed in Section 2. Section 3 overviews the related work. Section 4 describes the historical data used, including their sources. Locations of the addresses used in the past are analysed in Section 5, including a discussion. Survival analysis of the location lifetime is described in Section 6, including the discussion. Two use cases are described in Section 7. The work limitations are given in Section 8.

## 2. Problem of Retrospective IP Location

Retrospective IP geolocation is based on the availability of past IP location databases. Some past databases may be found by searching the historical content on the Internet. The success of the search highly depends on the targeted past dates (the range could be months to years) and how distant into the past it is. More databases can be found for recent dates and fewer for the distant past. The particular problems with the past databases availability are:

(i) Past databases are typically a part of other software, such as application installations or compressed files, such as software packages (e.g., Linux rpm). These data, which could be in all possible formats, need to be extracted and inspected in order to collect the past database. One needs to know or guess the application or software names that contain past geolocation databases.

(ii) Past databases are searched for a specific date of interest. However, the metadata of the files including the database do not correspond with the date the database was built. The reason is that the file date refers to the file-system storage time, which changes when the file is moved/copied (e.g., on an ftp repository). Thus, the file date is different from the database build date, with a possible difference of years. Dates incorporated into filenames are not also reliable, as they are not the date when the database was built. Therefore, it is not possible to search for the past databases by lower and upper date limits due to the file metadata and the database build date difference.

(iii) For some database formats, such as the common CSV, it is not possible to obtain the database build date as there is no such information stored in the database itself. Therefore, the database creation date is completely lost as time passes. The result is that many databases that may have been found cannot be used at all due to large date inaccuracies.

**Figure 1:**
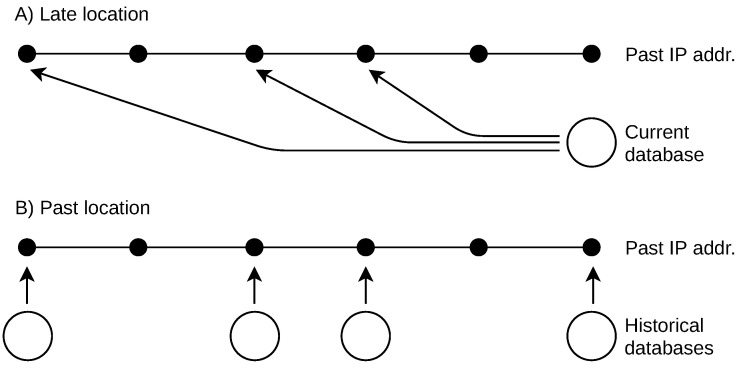
Approaches to retrospective IP geolocation due to missing past databases. (**A**) Naive approach with the current database used. (**B**) Past approach with a limited set of past databases used. The past databases should be close to the date of the past IP address use.

These historical data problems result in missing past databases. There are two basic approaches to retrospective IP location, as shown in Figure 1. The first is a naive solution that the past IP addresses are located by a current geolocation database. In this work, I refer to this naive approach as “late location”. The second approach uses an incomplete list of past databases to locate addresses used in the past. I refer to this approach as “past location”.

## 3. Related Work

A recent major comparison of IP location databases was done by Gharaibeh et al. [10]. They deal with the location accuracy of the addresses that belonged to router interfaces. Their ground truth included about 16,500 addresses. The evaluated geolocation databases were MaxMind GeoLite2 (free), IP2location DB11.Lite (free), MaxMind GeoIP2 (commercial), and Digital Envoy NetAcuity (commercial). The ground truth of router interface addresses was created by decoding the location hints from the router hostnames. The particular router addresses were collected from the RIPE Atlas built-in measurements. Specifically, the traceroute data were used to collect the addresses of the routers, which were within a distance of 50 km to the Atlas probes. The Atlas probe coordinates were used as reference locations. The work results were that the Digital Envoy NetAcuity had the best accuracy at the city level, followed by MaxMind GeoIP2 and GeoLite2. The latter databases performed similarly. The authors also used 692,000 addresses to study the inconsistency between the databases at the city level. The result was that at least 29% of the addresses across the databases had a city-level disagreement. A pairwise comparison of the MaxMind databases (free and commercial) showed that they were similar. There were 68% of the addresses with the same coordinates, and about 11% of the addresses had a distance difference of over 40 km. The authors also justified the usage of the 40 km limit for the city level, as this value is commonly used for location accuracy evaluation. In general, the databases may return different coordinates for the same city. These coordinates point, for example, to the organization headquarters with their own delegated address space or to the city cultural/geographical center. To justify this limit, the authors examined the distance between the coordinates from different databases. These coordinates were returned for the same cities. The result was that 99% of the same-city coordinates were within a distance of 40 km.

The active location is used to populate the databases, along with other methods, such as data mining and device self-reporting. The recent work of Du et al. [11] has dealt with active location using the RIPE Atlas platform called IPmap. A single-radius engine was used to deliver the location of IP addresses. The latency measurements approximated the geographical distance between the located device and the Atlas probe, whose coordinates were taken as the ground truth. The cities around the geographically closest Atlas probe were ordered, and the most probable city was used as the location result for the IP address. The measurements were done only by a selected set of Atlas probes to reduce the Internet traffic and location delay. A possible triggering of a DoS attack alert was mitigated this way. The ground truth used covered 968 addresses of which 870 were located. Eighty percent of them were located within the city-level distance (40 km) to the correct location.

Other work combined different location approaches to improve the accuracy. Scheitle et al. [7] used the hints in domain names and latency measurements to locate the routers. There was a location match in these two methods for 45,000 IPv4 addresses and 5000 IPv6 addresses. This way, they proposed an efficient way to establish a ground truth. Zhao et al. [3] combined a location database and latency measurements to reduce the number of NA (not available) locations. The work was based on a set of classifiers. The result was a location accuracy of 99% at the the province level and 82% at the city level. The results were obtained for places in China. The authors also mentioned a non-standard application of IP location to broadcast important information, for example, issued by the government. The information is displayed similarly to online adverts. The broadcasting range may be limited to users from specific cities, thus introducing a new channel for message delivery.

IP location accuracy suffers from constant changes in the Internet space. Padmanabhan et al. [12] studied the reasons behind the changes in the IP address space and consequently changes in their locations. The RIPE Atlas was used in this work as the ground truth. The ground truth IP addresses were obtained from the periodic connections of the Atlas probes to the controller. The authors worked with 3038 IPv4 probes over 12 months. The result was that ISPs assigned IP addresses to probes in a periodical manner, probably due to security reasons. Work by Almohri et al. [13] focused on the IP address assignment. They attempted to predict the next assigned address, which may be used to improve the location accuracy. The previous address location could be immediately assigned to the new address. This way, the negative effect on the address reassignments could be mitigated. The work particularly dealt with the IPv4 addresses used by cloud service providers, which were the Amazon Web Services and Google Cloud Platform. There were around 4000 addresses used. The prediction success of the next assigned address was around 90% for the first three address bytes, which is sufficient for IP geolocation.

There are many applications of IP geolocation, such as where the device locations may be needed retrospectively. These include address reputation [14], phishing mitigation [15], credit card fraud [16], and forensic investigation [17].

## 4. Collection of Historical Data

The historical data used in this work consist of IP addresses used in the past, their past locations, and the previous versions of a geolocation database. The addresses used in the past and their locations were processed from the RIPE Atlas archive [18]. This archive stores information about fixed devices, which are measurement probes. These probes are distributed around the world in different network environments. I use them as the historical ground truth since their past descriptive information is available, including IP address, operational status, and location. The archive starts in 2014 and is available up to 2021 as of the date of this work [19].

I processed the archived ground truth to avoid bias caused by invalid data, including possible faulty addresses. The Atlas software automatically stores the probe addresses when they periodically connect to the Atlas controller. The probe operational status is also automatically set by the success of these connections as Connected, Abandoned, Disconnected, and Never Connected. I excluded probes that were not in the status Connected at a given past date. This way, only the IP addresses that were truly active in the past were considered. The probes without a location specified in the past were also excluded. For example, the original number of ground truth probes on 2 March 2021 was 33,655, and it was reduced by this processing to 11,523. There were also some missing or invalid networking data (e.g., duplicated addresses). This further reduced the number of usable probes on that date to 9781. The final reduction resulted in about one-third of the original data for each year covered.

The original and reduced ground truth data over the years are shown in Table 1. The ground truth probes had IPv4, IPv6, or both addresses, as shown by the numbers. There were large differences in the number of usable probes between the years, mainly towards 2014, which was the start of the archive. The large difference in the number of probes could cause an inconsistency in the address space coverage. Therefore, I did not use the first two years (2014, 2015) of the ground truth for the IPv4 address historical analysis. For IPv6 addresses, I considered the years from 2018. In total, approx. 51 thousand IPv4 addresses and approx. 17 thousand IPv6 addresses were used.

**Figure 2:**
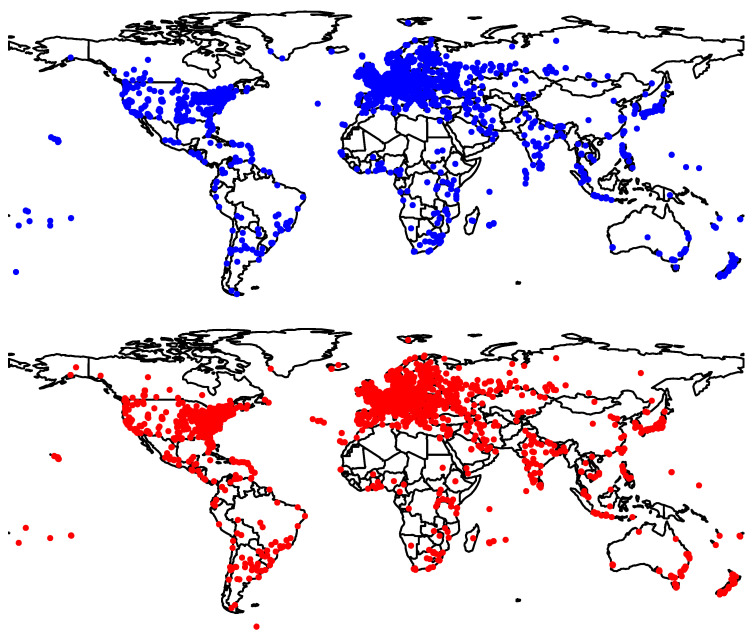
Ground truth global distribution in 2016 (blue) and 2021 (red).

Figure 2 shows the global geographical distribution of the ground truth probes. The majority of the ground truth was in Europe and North America.

The historical data include past versions of a geolocation database. I used the past versions of the MaxMind GeoLite2 City database. This database is commonly shipped as a part of various software and operating systems. This makes some of their past versions reachable in the respective software archives compared to other databases, which are not archived. These particular sources were used:

- Archives of UNIX and Linux OS updates [20]. The historical data were obtained from the past versions of software packages (e.g., rpm, deb).
- Web content archive provided by the Wayback Machine [21]. The historical data were obtained from the snapshots of pages’ past content, mainly in the MaxMind domain.
- Research datasets, such as Harvard Dataverse Repository [22].
- Installations of web servers that use IP geolocation, such as WordPress.
- GitHub and GitLab and other general software repositories. The data were obtained from the archived software development versions.

As discussed in Section 2, there is an issue with the database dates as the file metadata date is not the date when the database was built. Accurate dates of the past database are needed for past address locations and analysis. A single inaccurate date may give wrong results or wrong estimates of the location lifetime for many addresses. I also used the database date to link it to the correct day of the past ground truth. The MaxMind databases are stored in the MaxMind DB File Format (mmdb), which holds the “epoch” value [23]. I used this epoch as the genuine database building date. For databases that are stored in other formats, such as the common CSV, obtaining the genuine date is not possible.

The past databases were linked to the past ground truth with a prerequisite of the same relative difference between the database dates. The best match was found to be in March and April of each of the covered years. The databases complying with this prerequisite were dated to 3 April 2014, 3 March 2015, 3 March 2016, 7 March 2017, 4 April 2018, 15 April 2019, 3 March 2020, and 2 March 2021. The past ground truth comes exactly from these same dates, starting from 2016. The used past databases and Atlas archives are listed in Table A1.

The survival analysis is based on the duration of address locations over past databases. The databases store the locations for groups of addresses, delimited by a network range, for example, /24 for IPv4 addresses and /48 for IPv6 addresses. To extract the IP addresses, the CSV database version was used (the build date was obtained by direct download of the linked binary mmdb alternative of the same database). The database blocks are described as (simplified) network address/range, geoname_id, and coordinates. The geoname_id property refers to another CSV file storing the place textual descriptions. An example record for IPv4 is 71.195.26.0/23, 5037649, (45.0196, −93.2402), where the place geoname_id refers to Minneapolis, US. An example for IPv6 is 2a0f:9400:8008::/48, 3163392, (62.4684, 6.3427), where the place ID refers to Alesund, Norway. The CSV files used to derive the IPv4 and IPv6 addresses are listed in Table A2 as ‘source files for lifetime end’. I selected the locations linked to the first IP address of each block in the database dated to 13 April 2021. These IP address locations were further checked for their change in the closest database dated to 5 April 2021. The changed address locations were used in the survival analysis only as their lifetime end date was known this way. There were 421 k IPv4 and 47 k IPv6 addresses with the known location end date. In total, 38 past databases were used in the survival analysis. Table A2 lists the dates of the past databases used.

## 5. Retrospective IP Address Location

### 5.1. Late Location

**Figure 3:**
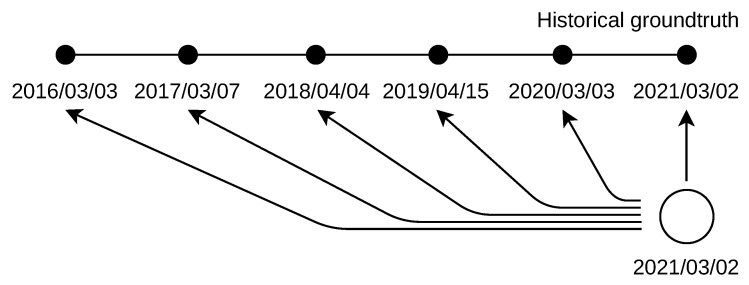
Late IP address location by the most current database. Past IPv4 addresses were dated from 2016. Past IPv6 addresses were dated from 2018.

Late location deals with the naive approach to handle missing geolocation databases. The IP addresses used in the past were located by the most current database as shown in Figure 3. The database used was dated to 2 March 2021. The ground truth was dated from 3 March 2016 to 2 March 2021 (day is first in the dot format; day is last in the reversed slash format, which is used for clarity in the figure).

**Figure 4:**
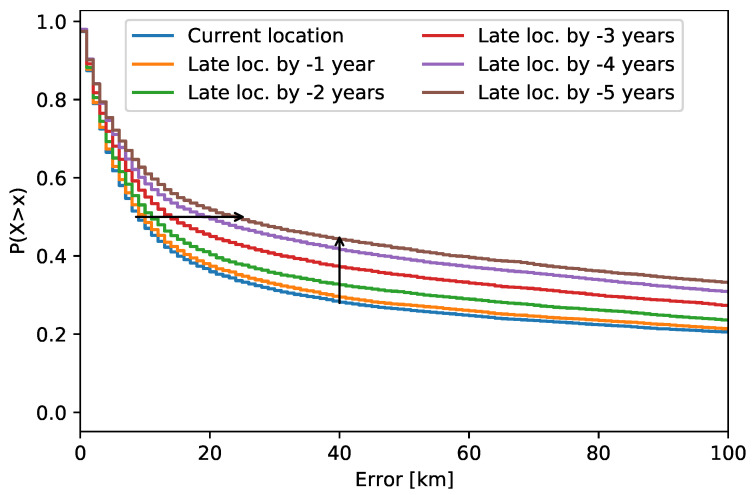
Changes in late location of IPv4 addresses over the years. The arrows show the changes in the location error median and in the percentage of locations outside the city level.

Figure 4 shows the probability of IPv4 address location error greater than *x* km, P(X>x)=1−P(X≤x). The error is the distance between the ground truth location and the estimated location. The “current location” curve describes the error for the addresses dated to the same date as the database build. It therefore shows the minimal location error. There was a constant error increase with the number of past years. The median location error increased by a minor value of up to 3 years to past; later, the increase was larger. The percentage of the locations outside the city level (40 km) changed with relatively small steps that were different across the years. The numerical values of the analysis are given in Table 2. These data also include the percentage of NA (not available) locations, which are the locations not returned by the database. For IPv4 addresses, there were no or a negligible number of NA locations over the years (below 0.07%).

IPv6 addresses showed a significant number of NA locations, which was approx. 7%. Their presence introduces a bias in the analysis, as the results indicate a better accuracy. This is true from the typical application point of view when all IP addresses are located and missing locations decrease the value of the system output. Consequently, with many NA locations, the system might have a good accuracy, but with an overall poor value.

**Figure 5:**
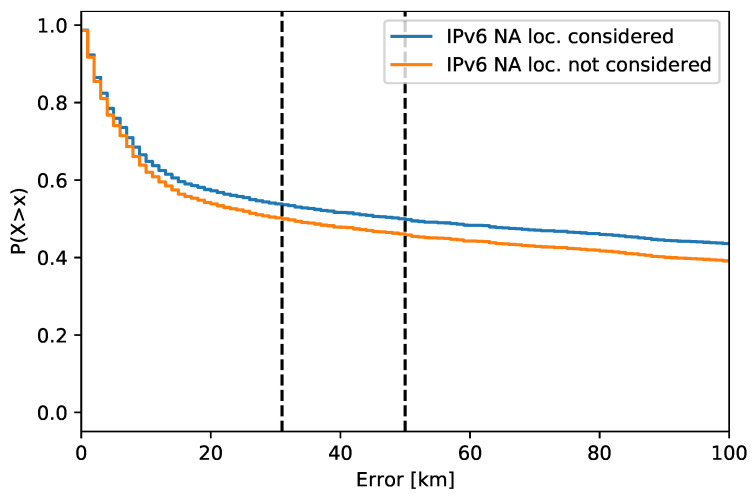
Effect of NA locations substituted by the maximal error observed for the ground truth in 2021.

The effect of NA locations on the accuracy in 2021 is shown in Figure 5. The red curve shows the accuracy when NA locations are not included. The blue curve shows the location accuracy with NA locations substituted by the maximal error observed for the ground truth in 2021. This is, in my opinion, an appropriate interpretation of the NA data from the application point of view. Moreover, the inclusion of NA locations is crucial when the accuracy is compared over different sets. The sets with a higher number of NA data are favored, as only more accurate locations are present. The original and updated results for IPv6 addresses in Table 2 show different values for the median location error. The relative differences over the years are about the same.

### 5.2. Past Location

**Figure 6:**
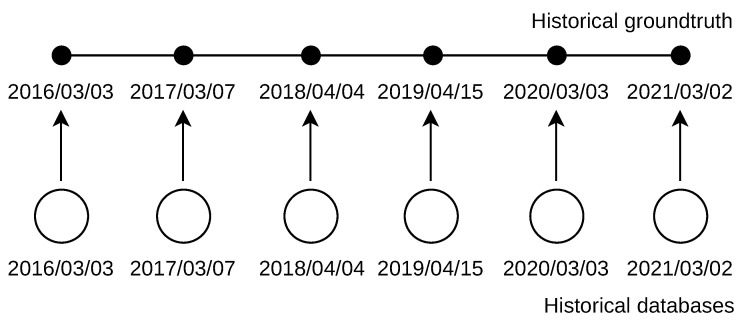
Past IP addresses location by historical databases. IPv4 addresses were dated from 2016. IPv6 addresses were dated from 2018.

With past location, the addresses used in the past were located by a set of past addresses. These databases were dated exactly to the same day as the addresses’ past use, as shown in Figure 6. The databases used were dated from 3 March 2016 to 2 March 2021 (the day is first in the dot format; the day is last in the reversed slash format, which is used for clarity in the figure).

**Figure 7:**
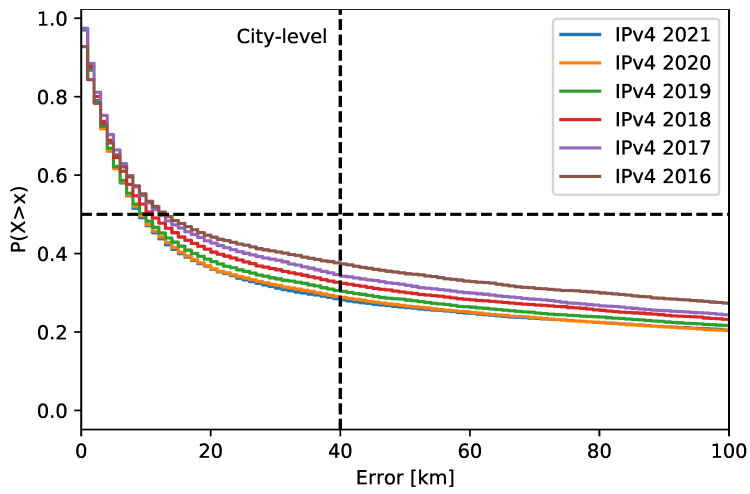
Past location of IPv4 addresses over the years. The dashed lines highlight the location error median and the percentage of locations outside the city level.

Figure 7 shows the probability of IPv4 address location error greater than *x* km. The median location error was higher in the past years, but only by a small value. However, the percentage of locations outside a city showed greater improvements over the years. This indicates that IP geolocation is improving over the years, especially at the city level, despite the constant changes in the address space. The numerical data for both IPv4 and IPv6 are given in Table 3. The number of NA locations for IPv4 addresses was again negligible (below 0.04%). On the other hand, their number was high for the IPv6 addresses, with percentages similar to the late location. The median error for IPv6 addresses varied across the years. The same was true for the percentage of locations outside the city level, which were about twice the value for IPv4 addresses.

The raw data used in the analysis are available in the format listed in Table A3.

## 6. IP Address Location Lifetime

Geolocation databases store locations for IP addresses. I apply survival analysis to these locations to investigate their lifetime. The location lifetime is the duration of the unchanged location over past geolocation databases. The survival function S(t)

(1) $$S\left(t\right)=P\left(T>t\right)=1-F\left(t\right)=\int_{t}^{\infty }f\left(u\right)du$$

gives the probability of a location surviving past time *t*, *T* is the random variable expressing the address location duration over past databases, and F(t) is the cumulative distribution function for t∈[0,∞), F(t)=P(T≤t). *T* is known to be within the *i*th interval $T_{i}\in \left(L_{i},R_{i}\right]$, where $L_{i}$ is the left interval limit and $R_{i}$ the right interval limit, i={1,⋯,N}.

**Figure 8:**
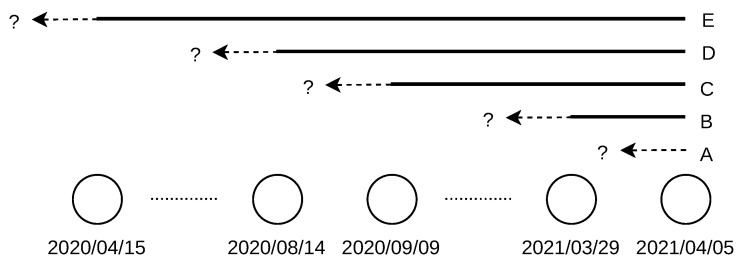
Interval censoring. A—address location changed within a week to past; C—address location changed between 14 August 2020 and 9 September 2020; E—address location changed before 15 April 2020.

The location lifetime *T* is expressed in days. The intervals of *T* are open from the left, $T_{i}>L_{i}$, as the location change had to happen before the past database date, which was used to obtain $L_{i}$. The intervals are closed from the right, $T_{i}\leq R_{i}$, as the location change had to happen after or on the database date, which was used to obtain $R_{i}$. For example, a location changed between 14 August 2020 and 9 September 2020, as the change was not observed in the database dated to 9 September 2020 (the location was “alive” on this day), but it was already observed in the database dated to 14 August 2020 (the location was “dead” on this day), as shown in Figure 8.

The location change is right-censored for cases when it was not observed until the last (most distant) historical database date. There are no left-censored data, as all location durations ended at time $E\in (L_{e},R_{e}]$. The interval of *E* and intervals of $T_{i}$ set the doubly interval censored data. The problem of doubly censored data is approached by the reduced likelihood and use of the maximal intervals for $T_{i}$ [24]. Therefore,

(2) $$T_{i}\in \left(\tilde{L_{i}},\tilde{R_{i}}\right]=\left(L_{i}-R_{e},R_{i}-L_{e}\right]\;,$$

where $R_{e}$ is set to eight as all locations ended between 13 April 2021 and 5 April 2021. $L_{e}$ is set to zero as 13 April 2021 is the database date used to derive the addresses. The last interval $T_{i}\in \left(355,\infty \right)$ indicates that the address location was alive (the same) before 15 April 2020; thus, the address location start was not observed. I assume that there cannot be two historical databases dated to the same date, and thus $\tilde{L_{i}}\neq \tilde{R_{i}}$, which states that any of the location changes cannot be observed on the exact day. I also assume that the interval censoring is non-informative, i.e., that it is independent of the likelihood of the location change. This means that the censored locations would have the same distribution of changes as if they were exactly observed.

Some locations changed with a low difference between the previous and new coordinates, such as (35.7298, 139.6347) to (35.69, 139.69), a distance of 7 km. There were also some changes that repeated periodically, thus alternating between a set of coordinates over time. To mitigate these false observations, I used a limit of 40 km distance between the coordinates to detect the true city-level change [10]. It was also possible that a past database did not have a record for an IP address; therefore, its location was NA. The first observation of the NA data was considered as the location change, thus ending the address location lifetime.

The definition of the address blocks in the past databases might have changed during the years. I worked with the addresses falling into different blocks the same way as with other addresses. This effectively means that if the address block was changed, the investigated address location was set to the new block location. Therefore, there were not any right-censored locations due to their dropping before the last observation time (note that there are right-censored data after the last observation time).

**Figure 9:**
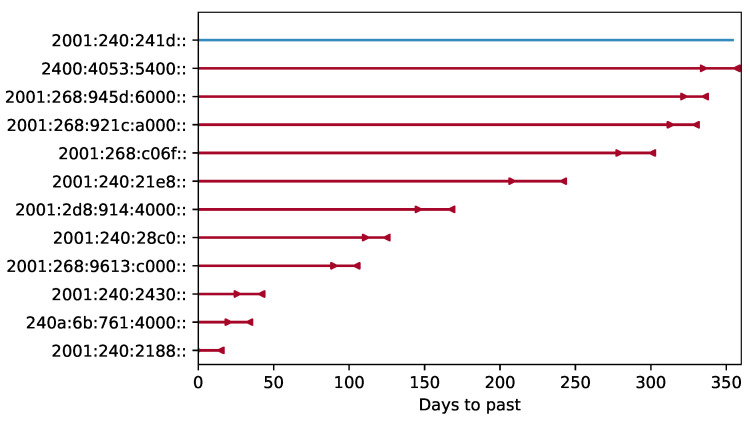
Sample interval censoring of IPv6 address location lifetime. First addresses of IP blocks are shown.

A sample of the interval-censored data for IPv6 addresses is shown in Figure 9. The arrows on the red lines delimit the intervals of location change. The blue lines show the cases when the location change was not observed during the covered period, that is, over one year.

Table 4 shows the interval-censored survival data in days to pass for IPv4 and IPv6 addresses. The column “removed” gives the number of locations changed during the interval. All location changes are censored, which is shown in the next two columns as observed =0 and censored = removed. The column “at_risk” gives the number of unchanged locations. There are the same intervals for IPv4 and IPv6 addresses. The last interval is right-censored.

I used the iterative Turnbull Estimator to estimate the survival function $\hat{S}\left(t\right)$, which handles the interval of censored data via maximum likelihood computation. The estimator [25] considers $\tau_{0}<\tau_{1}<\cdots <\tau_{m}$ times that include interval limits $L_{i}$ and $R_{i}$, $\tau_{0}=0$. A weight $a_{ij}$ is defined for *i*th observation and j={1,⋯,m} as

(3) $$a_{ij}=\left\{\begin{array}{cc} 1 & \left(\tau_{j-1},\tau_{j}\right]\in \left(L_{i},R_{i}\right]\\ 0 & \mathrm{otherwise} \end{array}\right.\;,$$

which shows whether the location change during $\left(L_{i},R_{i}\right]$ could have happened at time $\tau_{j}$. The initial guess of $S(\tau_{j})$ may be obtained by the Kaplan–Meier non-parametric estimator [26] as

(4) $$\hat{S}\left(t\right)=\prod_{t_{i}\leq t}\frac{n_{i}-d_{i}}{n_{i}}\;,$$

where $n_{i}$ is the number of same locations at risk up to the right limit of interval $R_{i}$, and $d_{i}$ is the number of locations that changed during $\left(L_{i},R_{i}\right]$.

The mass within the interval $\left(\tau_{j-1},\tau_{j}\right]$ is

(5) $$p_{j}=S\left(\tau_{j-1}\right)-S\left(\tau_{j}\right)$$

and the number of changed locations at $\tau_{j}$ is estimated as

(6) $$d_{j}=\sum_{i=1}^{n}\frac{a_{ij}p_{j}}{\sum_{k=1}^{m}a_{ij}p_{k}}\;.$$

Finally, the number of locations at risk at $\tau_{j}$ is computed as

(7) $$Y_{j}=\sum_{k=j}^{m}d_{k}\;.$$

The process of Equations (5)–(7) is repeated until the new survival function estimate is close to the previous S(t) for all $\tau_{j}$. The software [27] was used for this specific calculation.

**Figure 10:**
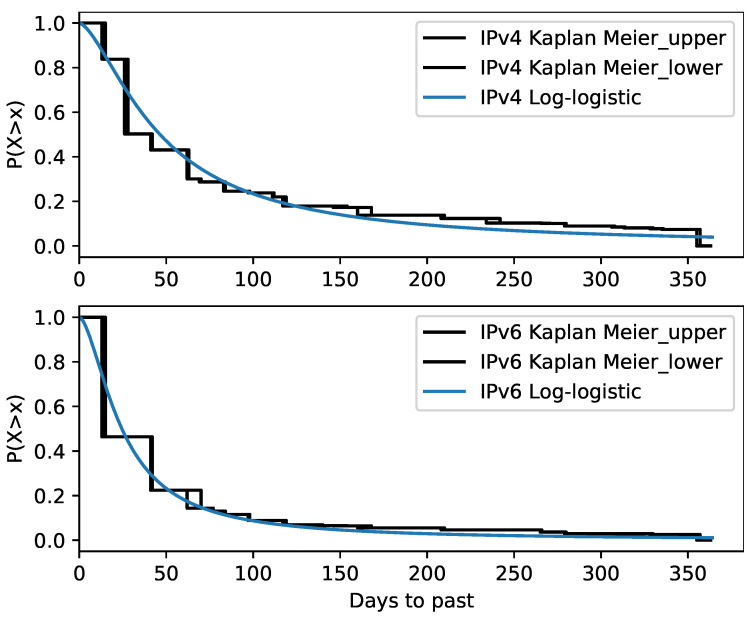
Estimation of address location lifetime. The approximated parametric model is also shown.

Figure 10 shows the estimate of the survival function for the locations of IPv4 and IPv6 addresses. The upper and lower survival boundaries for each interval are shown. The last bounding box is not defined due to right censoring.

The non-parametric survival function was approximated by the parametric Log-logistic model

(8) $$S\left(t\right)={\left(1+{\left(\frac{t}{\alpha }\right)}^{\beta }\right)}^{-1}\;,$$

where α is the scale parameter and β is the shape parameter. The model was chosen based on the best AIC score among the common parametric models. The model fit for both IPv4 and IPv6 is shown in Figure 10. The fitted parameters are listed in Table 5.

The survival analysis shows that IPv4 address locations may be considered as stable in time with respect to IPv6. The median location lifetime duration for IPv4 addresses is about 46 days and 24 days for IPv6 addresses.

The raw data used in the analysis are available in the format listed in Table A4.

## 7. Application Use Cases

Two use-cases with the results applied are described here. The first deals with the geotargeted online content, and the second deals with identity theft prevention.

### 7.1. Application of Late Location

**Figure 11:**
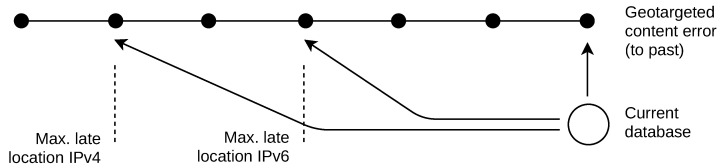
Geotargeted content use-case. Past viewers’ locations are used.

Use case: An e-shop system works with geotargeted content. The web pages modify their content (popups, text, displayed items, etc.) based on the viewers’ locations and create links dynamically to redirect them [28,29]. The geotargeted content display and redirection actions are defined by a set of rules. There are errors related to geotargeting (e.g., page crashes), and these were found to be repetitive over time. The errors are linked to viewers who access the system from specific cities. For example, a page error happens when a user from the city A tries to load it. We want to know all cities from which the users accessed the system during each error reported to find the links between the locations, geotargeted content, and rules. An archive of the users’ IPv4 and IPv6 addresses is available from the system log. The question is how far into the past the currently installed geolocation database can be used to locate the viewers’ addresses without a significant accuracy drop, as shown in Figure 11. A minor accuracy drop is acceptable as the links between the locations and page errors are tested. Seeking past databases for this purpose is not desired because of the extra time and resources required.

Result application: According to Table 2, the percentage of locations outside the city level grows with each year of late location. The median error increased after three years to the past by 5 km for IPv4, which is a low value. It is therefore acceptable to use the current database for the IPv4 addresses, which were used up to three years ago. However, the median error for IPv6 increased by 58 km after three years to the past, which makes the late location untrustworthy. It is therefore acceptable to use the current database to locate IPv6 addresses, which were used up to one year ago.

### 7.2. Application of Location Lifetime

**Figure 12:**
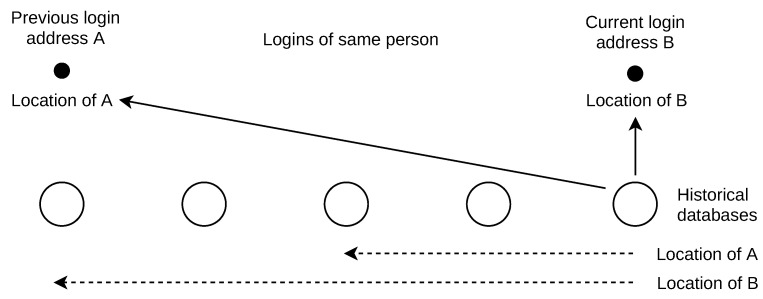
ID theft prevention use-case. The time difference between current and previous login (hours) is not in relation to the time difference between past databases (weeks).

Use case: An e-commerce merchant stores the previously used credit card(s) details for easy one-click payments by customers. The merchant wants to implement identity theft protection to minimize fraud losses and chargebacks. On the other hand, the protection should not impact revenues by not letting customers complete the payment smoothly. Therefore, a trade-off between a smooth shopping experience and improved security has to be resolved. ID theft protection is implemented by two-factor authentication initiated only on justified suspicions. The places of person’s subsequent logins (given the credentials used) are used to calculate the person’s travel speed [30], as shown in Figure 12. The maximum travel velocity of 400 km/h is used to detect the logins that are not possible for a single person [31]. If the person’s velocity is above the threshold, one-click payments are verified by two-factor authentication. If the verification fails, the stored credit card may be removed from the merchant’s system to reduce the risk of fraud (the customer needs to enter the card details manually).

The IP address location lifetime is used to assess the confidence in the maximum velocity violation. There are many false violations, as some devices frequently change their IP addresses. In such cases, the previous and current login places can be far apart even if the person does not travel. This is common for cellular devices with data plans, as their addresses change often, e.g., within hours [32]. These addresses are used at different locations, which are consequently changed in the geolocation database (typically by reports of these GPS-enabled cellular devices). As a result, the location lifetime for these addresses is short. This is different compared to broadband and fixed-device IP addresses [12]. Specific locations should be excluded from the lifetime observations, such as the country geographical centers and organization postal addresses stored in the WHOIS database [33]. For justified two-factor authentications, we want to exclude address locations that change often.

Result application: The time difference between current and previous logins from addresses A and B is one hour. The addresses are located at places 500 km apart (larger time and distances might be typical in ID theft). Past databases were inspected: the location for address A changed 140 days ago, and the location for address B changed 14 days ago. The survival probability for location of address A is $S\left(140\right)={\left(1+{\left(\frac{140}{46.38}\right)}^{1.55}\right)}^{-1}\approx 15\%$. This is a rarely long duration (only 15% of durations are longer); thus, it is not likely a cellular address. On the other hand, the survival probability for location of address B is $S\left(14\right)={\left(1+{\left(\frac{14}{46.38}\right)}^{1.55}\right)}^{-1}\approx 86\%$. This duration is common (86% of location durations are longer), and we are not confident that this address is not cellular. The two-factor authentication is not initiated and the suspicion is weak. The values are given for IPv4 addresses. By the parametric survival, the IPv4 and IPv6 lifetime can also be compared mutually (first login from IPv4 and second login from IPv6 address).

## 8. Work Limitations

This work has limitations in terms of the results applicability. The ground truth used for late and past locations covers fixed probes. Therefore, the results dealing with location accuracy in Section 5 are not valid for mobile devices. The results dealing with address lifetime in Section 6 are independent of the device type and thus valid for both mobile and fixed devices. The ground truth probes were not evenly distributed across the world. Hence, the results in Section 5 are mostly descriptive for the addresses from Europe and North America, where the majority of the ground truth was located. The locations of the RIPE Atlas ground truth are irreversibly obfuscated by up to one kilometer [34]. Considering the error distances in IP geolocation, units of kilometers can be considered as negligible. The work of [10] thoroughly analyzed the ground truth validity, and only 19 probes were found to have coordinates wrong as set to the country’s center. Another couple of probes with wrong locations were found by analyzing the communication latency. I assume that this low number of probes is negligible for the purpose of historical analysis, as it was 0.3% of the ground truth in 2017. The locations were delivered from the historical MaxMind Geolite2 City databases. Therefore, the results are valid for such a database. The justification for using this database in this work is given in Section 4.

## 9. Conclusions

This work has the following conclusions: Late location of IPv4 addresses by three years shows only a minor decrease in accuracy. This is not true for IPv6 addresses, where the late location of only one year shows a significant decrease in accuracy. The survival analysis shows that the database-stored location lifetime is different for IPv4 and IPv6 addresses. The median location lifetime for IPv4 addresses is about twice as long as the IPv6 location lifetime. The applied interval censoring allows a way to handle the problem of missing past geolocation databases. The fitted parametric lifetime model estimates the duration of address locations.

The results were applied in two use cases dealing with geotargeted content and ID theft prevention. Past viewers’ locations were used in the first use case. The results of the work show how far into the past the system may locate the viewers using a current geolocation database without a significant drop in accuracy. The address location lifetime was used in the second use case. The work results estimate the confidence in IP address location.

All historical and processed data used in this work were made available for reproducibility and further research [9]. The work might be repeated after five years to extend the knowledge, mainly for IPv6 addresses.

## Figures and Tables

**Table 1:** Historical ground truth data over years.

	2016	2017	2018	2019	2020	2021	∑
Probes org.	17,313	21,227	25,167	27,867	30,854	33,655	
Probes red.	7714	7793	8688	8802	9263	9781	
IPv4 addresses	7600	7675	8519	8598	9025	9470	50,887
IPv4 year used	✓	✓	✓	✓	✓	✓	✓
IPv6 addresses	3006	3189	3652	3920	4345	4650	16,567
IPv6 year used	NO	NO	✓	✓	✓	✓	✓

‘org.’ refers to original ground truth data, ‘red.’ refers to the reduced data. The address numbers are after the reduction.

**Table 2:** Late location of IPv4 and IPv6 addresses.

	−5 y	−4 y	−3 y	−2 y	−1 y	Cur.
IPv4						
Location median error [km]	24	20	14	11	10	9
Not available ^a^ [%]	-	-	-	-	-	-
Locations outside city level ^b^ [%]	44	42	37	33	29	28
IPv6						
Location median error ^c^ [km]	-	-	76	50	33	31
Not available [%]	-	-	8	7	7	7
Location median error ^d^ [km]	-	-	108	69	50	50
Locations outside city level ^b,d^ [%]	-	-	60	55	52	52

^a^ NA locations for IPv4 are none or negligible (below 1‰); ^b^ Location error over 40 km; ^c^ NA locations not considered; ^d^ NA locations considered.

**Table 3:** Past location of IPv4 and IPv6 addresses.

	2016	2017	2018	2019	2020	2021
IPv4						
Location median error [km]	13	12	11	10	9	9
Not available ^a^ [%]	-	-	-	-	-	-
Locations outside city level ^b^ [%]	37	34	32	30	29	28
IPv6						
Location median error ^c^ [km]	-	-	84	61	78	31
Not available [%]	-	-	7	7	7	7
Location median error ^d^ [km]	-	-	107	78	100	50
Locations outside city level ^b,d^ [%]	-	-	63	58	61	52

^a^ NA locations for IPv4 are none or negligible (below 1‰); ^b^ Location error over 40 km; ^c^ NA locations not considered; ^d^ NA locations considered.

**Table 4:** Survival tables for location lifetime of IPv4 (above) and IPv6 addresses (below).

R Int. Limit	Removed	Observed	Censored	Entrance	At_Risk
	0	0	0	421,033	421,033
15.0	38,470	0	38,470	0	421,033
21.0	11,429	0	11,429	0	382,563
28.0	56,585	0	56,585	0	371,134
…	…	…	…	…	…
343.0	2937	0	2937	0	34,090
357.0	708	0	708	0	31,153
363.0	1560	0	1560	0	30,445
inf	28,885	0	28,885	0	28,885
**R Int. Limit**	**Removed**	**Observed**	**Censored**	**Entrance**	**At_Risk**
	0	0	0	46,769	46,769
15.0	7099	0	7099	0	46,769
21.0	14,467	0	14,467	0	39,670
28.0	3528	0	3528	0	25,203
…	…	…	…	…	…
343.0	119	0	119	0	1298
357.0	31	0	31	0	1179
363.0	84	0	84	0	1148
inf	1064	0	1064	0	1064

**Table 5:** Fitted Log logistic model parameters. α also equals to median survival.

	Coef	Std. Error	Lower 95%	Upper 95%
IPv4 α	46.38	0.08	46.22	46.53
IPv4 β	1.55	0.00	1.54	1.55
IPv4 α	24.46	0.12	24.22	24.7
IPv4 β	1.67	0.01	1.66	1.69

## Data Availability

The reproducibility data are available at [9].

## Appendix A. List of Reproducibility Data

**Table A1:** Collection for historical analysis.

Past ver. of Geoloc. Database	Past Loc. and Addresses
20140403_GeoLite2.mmdb	
20150303_GeoLite2.mmdb	
20160303_GeoLite2.mmdb	20160303_Atlas.json
20170307_GeoLite2.mmdb	20170307_Atlas.json
20180404_GeoLite2.mmdb	20180404_Atlas.json
20190415_GeoLite2.mmdb	20190415_Atlas.json
20200303_GeoLite2.mmdb	20200303_Atlas.json
20210302_GeoLite2.mmdb	20210302_Atlas.json

**Table A2:** Collection of data for survival analysis.

Past Geoloc. Databases	Past Geoloc. Databases
20200415_GeoLite2.mmdb	20201209_GeoLite2.mmdb
20200421_GeoLite2.mmdb	20201215_GeoLite2.mmdb
20200505_GeoLite2.mmdb	20201222_GeoLite2.mmdb
20200512_GeoLite2.mmdb	20201229_GeoLite2.mmdb
20200518_GeoLite2.mmdb	20210105_GeoLite2.mmdb
20200527_GeoLite2.mmdb	20210112_GeoLite2.mmdb
20200603_GeoLite2.mmdb	20210119_GeoLite2.mmdb
20200609_GeoLite2.mmdb	20210122_GeoLite2.mmdb
20200616_GeoLite2.mmdb	20210126_GeoLite2.mmdb
20200630_GeoLite2.mmdb	20210202_GeoLite2.mmdb
20200707_GeoLite2.mmdb	20210209_GeoLite2.mmdb
20200714_GeoLite2.mmdb	20210218_GeoLite2.mmdb
20200721_GeoLite2.mmdb	20210223_GeoLite2.mmdb
20200814_GeoLite2.mmdb	20210302_GeoLite2.mmdb
20200909_GeoLite2.mmdb	20210310_GeoLite2.mmdb
20200915_GeoLite2.mmdb	20210316_GeoLite2.mmdb
20201027_GeoLite2.mmdb	20210323_GeoLite2.mmdb
20201110_GeoLite2.mmdb	20210329_GeoLite2.mmdb
20201124_GeoLite2.mmdb	20210405_GeoLite2.mmdb
Source files for lifetime end	
20210413_GeoLite2-Blocks-IPv4.csv	
20210413_GeoLite2-Blocks-IPv6.csv	

**Table A3:** Format of file 2016–2021_history.csv with processed historical data and sample.

Item	Value	Descripton
id	11299	Atlas probe ID
lat	51.5115	Atlas probe coord.
lon	7.4915	Atlas probe coord.
v4	37.24.156.234	Atlas probe IP
v6	2a02:908:4e3:8c40: …	Atlas probe IP
cntr	DE	Atlas probe country
adate	20180404	Probe activity date
v4latelocLat	48.7349	Current database (2021)
v4latelocLon	9.1521	
v4latelocCity	Stuttgart	
v4latelocCntr	DE	
v4latelocErr	331	
– values for v6 –		
v4pastlocLat	51.5208	Past database 20180404
v4pastlocLon	7.5184	
v4pastlocCity	Dortmund	
v4pastlocCntr	DE	
v4pastlocErr	2	
– values for v6 –		

**Table A4:** Format of file 2020–2021_lifetime.csv with processed survival data and sample.

Address	Ver	LatLon	DatabaseL	DatabaseR	L	R
24.172.103.136	v4	35.1873, −79.473	2021-03-10	2021-03-02	26	42
179.109.149.0	v4	−21.033, −41.939	2020-04-15	NaT	355	NaN
240a:6b:950…	v6	36.659, 139.822	2021-01-12	2021-01-05	83	98
2001:470:1f…	v6	51.2993, 9.491	2021-04-05	2021-03-29	0	15

L, R—in days; location lifetime ended between 5 April 2021 and 13 April 2021.
